# Artificial intelligence in obesity management: clinical evidence, translational gaps, and implementation priorities—a structured narrative review

**DOI:** 10.3389/fendo.2026.1955223

**Published:** 2026-09-03

**Authors:** Wenneng Liu, Jian Zhao, Dan Zhang, Chun Dang, Yaoheng Lu, Xiao Du

**Affiliations:** 1 Department of General Surgery, Chengdu Integrated Traditional Chinese Medicine and Western Medicine Hospital, Chengdu, China; 2 Department of Periodical Press/Chinese Evidence-Based Medicine Center, West China Hospital, Sichuan University, Chengdu, China; 3 Division of Gastrointestinal Surgery, Department of General Surgery, West China Hospital, West China School of Medicine, Sichuan University, Chengdu, China

**Keywords:** artificial intelligence, bariatric surgery, digital health, large language models, machine learning, obesity, weight management

## Abstract

Obesity is a chronic, relapsing disease that requires long-term lifestyle treatment, pharmacotherapy, and metabolic and bariatric surgery. Artificial intelligence (AI) is increasingly being studied across these pathways, but its clinical readiness varies substantially. This Scale for the Assessment of Narrative Review Articles (SANRA)-informed structured narrative review searched PubMed/MEDLINE, Embase, Scopus, ScienceDirect, and Google Scholar through 1 June 2026, primarily for literature published since 2015, with citation chaining used to identify earlier landmark studies. Evidence was appraised according to study design, validation, clinical utility, workflow integration, and demonstrated patient-level benefit. Direct patient-level evidence for explicitly AI-enabled obesity interventions remains limited and heterogeneous. The strongest weight or metabolic outcome evidence largely comes from multicomponent digital, automated or hybrid-care programmes, including studies in which no AI component was independently evaluated or in which diabetes and HbA1c constituted the primary clinical context and endpoint. These findings support digital-care delivery but should not be interpreted as evidence of an AI-specific therapeutic effect in obesity. AI-assisted drug discovery remains preclinical, while natural language processing of glucagon-like peptide-1 receptor agonist narratives can support signal detection but not causal inference or quantitative safety estimation. Phenotype-based treatment and a machine-learning-assisted genetic risk score suggest potential for responder stratification, but within the eligible evidence included in this review, no externally validated, prospectively implemented AI prescribing system was found to have demonstrated improved patient outcomes. In metabolic and bariatric surgery, AI may support risk prediction, operative workflow analysis, readmission stratification, weight-trajectory modelling, and digital follow-up; most studies, however, remain retrospective or internally validated and rarely assess calibration, actionable thresholds, or prospective impact. Large language models may assist education and drafting, but current evidence does not support unsupervised treatment or procedure selection. AI should therefore augment, not replace, clinician-led multidisciplinary obesity care. Translation will require independent external validation, prospective workflow evaluation, patient-centred outcomes, safety, fairness, data governance, and cost-effectiveness.

## 1 Introduction

Obesity is a chronic, relapsing and progressive disease and a major modifiable contributor to premature mortality and disability. It is closely associated with type 2 diabetes, cardiovascular disease, hypertension, dyslipidaemia, fatty liver disease and other metabolic complications (1). Management therefore requires long-term, multidisciplinary care integrating lifestyle intervention, anti-obesity pharmacotherapy and metabolic and bariatric surgery (2). Glucagon-like peptide-1 (GLP-1) receptor agonists and dual glucose-dependent insulinotropic polypeptide (GIP)/GLP-1 receptor agonists have substantially improved non-surgical weight loss. Contemporary metabolic and bariatric surgery remains highly effective for severe obesity and has a favourable perioperative safety profile, although procedure-specific complications and long-term nutritional and metabolic follow-up remain clinically relevant. Across treatment pathways, practical challenges include heterogeneous response, adverse effects or tolerability, adherence, affordability, access and continuity of long-term care (3, 4). AI-based methods are being investigated across each of these care pathways, although routine adoption remains limited outside selected structured digital programmes. Digital systems have been studied for food-image recognition, personalised feedback, automated reminders and remote monitoring; machine learning and natural language processing have been evaluated for drug discovery, patient narratives and pharmacovigilance; and surgical studies include perioperative risk prediction, operative-time estimation, video analysis, complication and readmission prediction, weight-trajectory modelling and digital follow-up. LLMs are also being evaluated for patient education, clinical question answering and procedure recommendations. These applications span very different evidentiary levels, from randomised patient-level trials to simulations, retrospective databases, *in vitro* experiments and hypothesis-generating text analyses (5–8).

Existing reviews have catalogued these applications, but technical feasibility, model discrimination, user acceptability and clinical benefit are often discussed without sufficient separation.

Recent obesity-specific syntheses have examined AI-enabled behavioural coaching and LLM or ChatGPT applications more directly. Hallock et al. (9). reviewed 21 studies of AI-enabled behavioural coaching platforms, whereas Suenghataiphorn et al. synthesised 33 studies of LLMs in obesity care (10) and Motevalli et al. examined 37 studies of ChatGPT applications in obesity management (11). These reviews identified promising applications but also emphasised heterogeneous study designs, short follow-up, variable accuracy, limited high-confidence evidence, ethical and equity concerns, and the continuing need for clinician oversight. Digital interventions frequently evaluate multicomponent care packages rather than an isolated AI effect; surgical models may report the area under the receiver operating characteristic curve (AUROC) without calibration, decision-curve analysis, external validation or workflow impact; and fluent LLM answers may not agree with real clinical decisions (12–14). The contribution of the present review is therefore not another catalogue of AI applications, but a cross-pathway assessment integrating lifestyle care, pharmacotherapy and drug development, metabolic and bariatric surgery, postoperative monitoring, and LLMs within a common clinical translational-readiness framework. This review therefore evaluates major AI applications in obesity management according to clinical translational readiness, emphasizing patient-level outcomes, validation, calibration, actionable thresholds, workflow integration, safety, fairness and prospective impact.

## 2 Review design, search strategy and study selection

### 2.1 Review design and SANRA-informed methodological framework

This structured narrative review examined AI across lifestyle intervention, digital weight management, anti-obesity pharmacotherapy, drug discovery, metabolic and bariatric surgery, postoperative monitoring and LLM-assisted communication. It was not designed to provide an exhaustive systematic review or pooled effect estimates. An approach informed by the Scale for the Assessment of Narrative Review Articles (SANRA) was used to strengthen the justification, aims, search description, referencing, scientific reasoning and presentation of clinically relevant endpoints; SANRA was not used to score individual studies (15).

### 2.2 Search strategy

The search was last updated on 1 June 2026. PubMed/MEDLINE, Embase, Scopus, ScienceDirect, and Google Scholar were searched for peer-reviewed studies published from 1 January 2015 to 1 June 2026. Earlier landmark studies were identified through backward and forward citation searching. Separate domain-specific searches addressed: (1) AI-enabled lifestyle and digital interventions; (2) anti-obesity pharmacotherapy, drug discovery, and pharmacovigilance; (3) metabolic and bariatric surgery; and (4) chatbot and large language model applications. Controlled vocabulary and free-text terms were combined for obesity, weight management, anti-obesity medications, metabolic and bariatric surgery, artificial intelligence, machine learning, deep learning, natural language processing, computer vision, predictive modelling, digital therapeutics, conversational agents, and large language models. Complete database-specific search strings, search dates, filters, and screening limits are provided in the **Supplementary Methods** (**Supplementary Search Strategies**).

### 2.3 Eligibility criteria and study selection

Eligible evidence included randomised and pragmatic trials, observational and real-world studies, model-development and validation studies, digital-health evaluations, natural language processing (NLP) and computer-vision studies, LLM evaluations, reviews, consensus documents and methodological guidelines. Primary application studies were eligible if they addressed obesity management or bariatric care, explicitly used AI or data-driven algorithms, and reported clinical outcomes, patient-centred outcomes, model performance, validation, implementation or translationally relevant feasibility. Non-AI digital or clinical studies were included only as contextual evidence, comparators, candidate-feature studies, or infrastructure linked to later AI applications; these studies were not treated as evidence of AI effectiveness. Weakly related reports, promotional materials, conference abstracts without substantive data, duplicates and studies with insufficient methodological information were excluded. Two authors independently screened titles and abstracts and reviewed potentially eligible full texts; disagreements were resolved by discussion or third-author adjudication. Priority was given to randomised, prospective, multicentre, externally validated and large real-world studies.

The searches identified 17,153 records across five electronic sources (PubMed/MEDLINE, Embase, Scopus, ScienceDirect, and Google Scholar) and 762 additional records through backward and forward citation searching. After removal of 1,682 duplicates, 16,233 unique records underwent title and abstract screening. Following exclusion of 14,907 records, 1,326 full-text reports were assessed for eligibility; 1,232 were excluded, leaving 94 publications for the qualitative synthesis. Because this was a structured narrative review rather than a systematic review, the diagram is presented as a descriptive account of study selection and does not imply full PRISMA compliance.

### 2.4 Data extraction and qualitative synthesis

Extracted information included study design, population or data source, AI method, comparator, endpoints, performance metrics, validation strategy, follow-up, patient-level outcomes, implementation setting and limitations. Owing to substantial heterogeneity, evidence was synthesized qualitatively rather than pooled. We distinguished technical feasibility, internal model performance, external validation or calibration, user acceptability, workflow integration, clinical decision support and demonstrated patient-level benefit.

For digital interventions, priority was given to weight change, metabolic outcomes, engagement, adherence and safety. Pharmacotherapy evidence was separated into drug discovery, patient-experience or pharmacovigilance analyses and responder stratification. Bariatric studies were assessed for risk prediction, video analysis, complications, readmission, weight trajectories and digital follow-up; LLM studies were assessed for accuracy, guideline concordance, readability, reproducibility, hallucination risk and suitability for supervised use. For lifestyle and digital interventions, evidence attribution was classified separately from study quality. Studies were treated as evidence from an explicitly AI-enabled intervention only when the primary report specified an AI component that was evaluated within the intervention. Studies were treated as contextual digital or automation evidence when no AI component was explicitly evaluated, when the intervention represented a multicomponent digital-care package without isolation of the AI contribution, or when the principal population and primary endpoint concerned an adjacent condition rather than obesity treatment. Classification as explicitly AI-enabled did not imply that the independent therapeutic effect of the AI component had been isolated.

### 2.5 Evidence appraisal for clinical translation

Evidence relevant to clinical translation was described across domain-appropriate dimensions, including study design and representativeness; prospective evaluation; external validation; calibration and decision-curve analysis where applicable; patient-level outcomes and follow-up; workflow integration; interpretability and human oversight; safety, fairness, privacy and regulatory considerations; and evidence of clinical impact. The synthesis reports the evidence profile, currently supported clinical role and unresolved evidence gaps for each application domain.

Direct patient-level evidence for explicitly AI-enabled obesity interventions remained limited. The strongest patient-level weight or metabolic outcome evidence in the broader technology-supported literature came from multicomponent digital, automated or hybrid-care programmes, which should be interpreted as contextual evidence for care delivery rather than evidence of an AI-specific therapeutic effect. Explicit AI involvement varied across these interventions, and the observed effects generally reflected multicomponent digital care rather than an isolated algorithmic treatment effect. Applications with clinically relevant targets but insufficient evidence of prospective clinical net benefit included perioperative risk and readmission models, operative-video analysis, long-term weight-trajectory prediction, dynamic postoperative monitoring and emerging AI-guided pharmacotherapy responder stratification. Some of these applications had retrospective, external-validation or prospective feasibility data, but routine use remained limited by varying combinations of incomplete calibration, limited independent validation, uncertain actionable thresholds and lack of evidence that model-guided actions improve outcomes. Earlier-stage applications should be distinguished by intended use: AI-assisted drug discovery provided mainly preclinical or early translational evidence; social-media and narrative-based pharmacovigilance supported signal detection and hypothesis generation; and generative nutrition or exercise tools, chatbots and LLM-based surgical decision support primarily supported feasibility, communication or clinician-reviewed drafting. Patient-facing or high-risk decision-support systems in these latter categories should not be used autonomously.

Reports of completed AI intervention trials were interpreted in relation to CONSORT-AI, whereas AI clinical-trial protocols, where applicable, were interpreted in relation to SPIRIT-AI. Early live clinical evaluations of AI decision-support systems were considered against DECIDE-AI, prediction-model studies against TRIPOD+AI and PROBAST+AI, and AI-based diagnostic-accuracy studies against STARD-AI. These frameworks guided qualitative appraisal and were not applied as formal study-level scores (14, 16–20).

## 3 Explicitly AI-enabled lifestyle interventions and contextual digital-care evidence

Lifestyle intervention remains the foundation of obesity care. To avoid attributing the effects of digital care to AI, we separated evidence from explicitly AI-enabled interventions from contextual evidence involving digital therapeutics, automation or hybrid care. Among the five representative patient-level studies, only eTRIP and AI-DPP explicitly evaluated AI-enabled interventions. Oviva Direkt and zanadio did not explicitly evaluate an AI component, while the study by Lee et al. principally evaluated diabetes management with HbA1c as the primary endpoint. The latter three studies were therefore retained only as contextual evidence concerning digital-care infrastructure, delivery and implementation.

### 3.1 Digital therapeutics, explicitly AI-enabled platforms and relevant automation

Among the five representative patient-level studies, eTRIP and AI-DPP explicitly evaluated AI-enabled interventions. Chew et al. evaluated 230 participants who completed a 1-week AI-assisted self-regulation intervention using food-image recognition, chatbot-guided assessment and personalised reminders. Overeating scores decreased by 0.32 (SD 1.16; *P* < 0.001), snacking scores by 0.22 (SD 1.12; *P* < 0.002), and physical activity increased by 1288.60 units (SD 3055.20; P < 0.001). The source article reported these as MET-min/day, although IPAQ-SF continuous scores are conventionally expressed as MET-min/week. This apparent source-level unit inconsistency should be interpreted cautiously. The single-arm, short-duration design and self-reported outcomes support feasibility rather than durable weight-loss efficacy (21). AI-DPP randomised 368 adults with prediabetes and overweight or obesity to a fully automated AI-led Diabetes Prevention Program or a CDC-recognised human-coached programme. At 12 months, the composite primary endpoint was achieved by 31.7% and 31.9%, respectively (risk difference, −0.2%; one-sided 95% CI lower bound, −8.2%), meeting the prespecified noninferiority criterion. Programme initiation was higher with AI-led care (93.4% vs 82.7%). These findings support scalable automated delivery, higher programme initiation and noninferior composite outcomes, but do not demonstrate clinical superiority over human coaching or isolate an AI-specific therapeutic effect (5).

Oviva Direkt, zanadio and the study by Lee et al. were retained as contextual rather than direct AI evidence. Oviva Direkt randomised 164 adults with obesity to a multicomponent digital therapeutic or usual care. At 6 months, mean weight loss was 5.29% with the intervention and 1.76% with control (estimated marginal mean difference, -3.53%; d = 0.80); 49% versus 4.8% achieved at least 5% weight loss. Objective weights were obtained using calibrated scales under video supervision. Limitations included lack of blinding, short follow-up, predominantly German recruitment, limited older-adult representation and the absence of a separately specified or evaluated AI component; the clinical effects therefore reflect the full digital therapeutic (22). Zanadio described 11,323 users with a body mass index (BMI) of 30–45 kg/m^2^ (mean age, 45.6 years; mean BMI, 36.6 kg/m^2^; 79.6% women). Among this cohort, hypertension, thyroid disease, spine or joint disorders and diabetes were reported in 42.3%, 32.9%, 30.4% and 17.8%, respectively. The 12-month programme combined education, diet and activity tracking, SMART goals, motivational support, professional chat and automated feedback. The primary study did not explicitly specify or evaluate an AI component. It demonstrated real-world reach, but the absence of a control group, reliance on self-report and limited longitudinal weight data preclude efficacy conclusions (23). Lee et al. randomised 294 Korean adults with type 2 diabetes in a 48-week multicentre trial of an integrated digital platform using connected devices and deep-learning food recognition. At 24 weeks, glycated haemoglobin (HbA1c) changed by -0.06% with usual care, -0.32% with the platform alone and -0.49% with the platform plus human feedback, with benefits maintained at 48 weeks. This provides adjacent metabolic and dietary-management evidence rather than direct evidence of AI effectiveness for obesity treatment; the primary outcome was HbA1c, and the population had type 2 diabetes (24).

Collectively, direct patient-level evidence from explicitly AI-enabled obesity interventions remains limited and heterogeneous. The strongest weight and metabolic outcome evidence largely concerns multicomponent digital, automated or hybrid-care programmes whose AI-specific contribution was not independently evaluated. Representative studies explicitly evaluating AI-enabled interventions are summarised in **Table 1**, whereas contextual evidence from digital, automated and adjacent metabolic-care interventions is presented separately in **Table 2**.

**Table 1:** Patient-level studies explicitly evaluating AI-enabled lifestyle interventions relevant to obesity care.

Study	Design and population	Intervention and AI components	Comparator and follow-up	Key outcomes	Main limitations and clinical interpretation
Mathioudakis et al. (2025) (5)	Phase 3 pragmatic noninferiority RCT; 368 adults with prediabetes and overweight or obesity at two US sites.	Fully automated AI-led Diabetes Prevention Program delivered through a mobile application and Bluetooth-enabled scale.	Remote CDC-recognised human coach-led DPP; 12 months. The composite endpoint required HbA1c <6.5% plus prespecified weight-loss, physical-activity or HbA1c-improvement criteria.	Composite endpoint: 31.7% with AI-led care versus 31.9% with human-coached care; absolute difference −0.2 percentage points (one-sided 95% CI lower bound, −8.2), meeting the prespecified noninferiority criterion. Programme initiation was 93.4% versus 82.7%.	The composite endpoint did not represent weight loss alone, and the trial compared complete programme-delivery models rather than isolating the AI component. Supports scalable automated delivery, higher programme initiation and noninferior composite outcomes, but not clinical superiority over human coaching or an independent AI-specific therapeutic effect.
Chew et al. (2024) (21)	Single-group pretest–posttest study; 230 completers with overweight or obesity in Singapore; 1-week intervention.	eTRIP: chatbot check-ins concerning eating triggers, local-food computer-vision recognition, meal logging, timed nudges and a meal stopwatch.	No comparator; outcomes assessed after 7 days.	Overeating score −0.32 (SD 1.16; P < 0.001); snacking score −0.22 (SD 1.12; P < 0.002); physical activity +1288.60 (SD 3055.20; P < 0.001), reported by the source as MET-min/day, inconsistent with the usual IPAQ-SF MET-min/week convention. Attrition was 8.4%.	Very short, single-arm study with self-reported outcomes and no objective weight-loss endpoint. Supports feasibility and short-term behavioural signals rather than sustained weight-loss efficacy or an isolated AI-specific therapeutic effect.

AI, artificial intelligence; CDC, Centers for Disease Control and Prevention; CI, confidence interval; DPP, Diabetes Prevention Program; HbA1c, glycated haemoglobin; IPAQ-SF, International Physical Activity Questionnaire–Short Form; MET, metabolic equivalent of task; RCT, randomised controlled trial; SD, standard deviation.

**Table 2:** Contextual patient-level evidence from digital, automated and adjacent metabolic-care interventions.

Study	Design and population	Intervention and digital/AI components	Comparator and follow-up	Key outcomes	Main limitations and clinical interpretation
Lautenbach et al. (2026) (22)	Decentralised RCT; 164 adults with obesity (BMI 30–45 kg/m^2^) in Germany.	Oviva Direkt: multicomponent digital therapeutic incorporating photo-based meal logging, digital education, goal setting, meal plans and automated feedback.	Usual care; 6 months. Weight measured with calibrated digital scales during supervised video calls.	Mean weight change: −5.29% vs −1.76%; adjusted between-group difference −3.53% (95% CI −5.16 to −1.91; d=0.80). At least 5% weight loss: 49.0% vs 4.8%.	Unblinded, Germany-only trial; 6-month retention was 76%, with limited representation of older adults. Supports patient-level effectiveness of the complete digital therapeutic but is not evidence of an AI-specific treatment effect.
Forkmann et al. (2022) (23)	Programme description and baseline real-world characterisation of 11,323 registered users with BMI 30–45 kg/m^2^.	zanadio: 6–12-month multimodal application providing education, nutrition and activity tracking, SMART goals, professional chat, device connectivity and automated reports or reminders.	No comparator; the publication described programme reach and baseline characteristics rather than longitudinal effectiveness.	Demonstrated national reach; mean age 45.6 years, mean BMI 36.6 kg/m^2^ and 79.6% women. No observed longitudinal weight-loss estimate was reported in this analysis.	Self-reported baseline data, selected registered users and no outcome comparator. Provides contextual evidence concerning digital programme reach and infrastructure, not weight-loss efficacy or an AI-specific therapeutic effect.
Lee et al. (2023) (24)	Open-label, multicentre, three-arm RCT; 294 adults with type 2 diabetes and overweight or obesity.	Integrated digital platform using connected devices and AI-based dietary management; one arm also received medical-staff feedback and intermittent personal CGM.	Routine care (n=99) vs platform alone (n=97) vs platform plus human feedback/CGM (n=98); 48 weeks.	HbA1c change at 24 weeks: −0.06%, −0.32% and −0.49%, respectively; benefits were maintained at 48 weeks. Digital arms had greater weight loss than routine care at 24 weeks.	The population had type 2 diabetes, HbA1c was the primary endpoint, and the intervention combined multiple digital and human-support components. Provides adjacent evidence regarding AI-supported dietary and metabolic management in diabetes, not direct evidence of an AI-specific treatment effect in obesity.

AI, artificial intelligence; BMI, body mass index; CGM, continuous glucose monitoring; CI, confidence interval; HbA1c, glycated haemoglobin; RCT, randomised controlled trial; SMART, specific, measurable, achievable, relevant and time-bound.

### 3.2 Chatbots, conversational agents, and generative AI tools

Chatbots, conversational agents and generative tools can support dietary advice, activity reminders, self-monitoring and education, but their evidence is substantially weaker than that of structured digital therapeutics. Most evaluations are small, short, non-randomised or technically focused (25).

Paola, an IBM Watson-based virtual coach, was evaluated in a 12-week proof-of-concept study of 31 adults aged 45–75 years. Preliminary changes in physical activity, diet and body composition and high acceptability were reported. Smartphone and technical barriers affected early engagement: approximately one-quarter of participants did not complete the first independent weekly check-in, whereas 12-week retention was 90% (26).

A different study examined SlimMe, a system that combined Dialogflow, LINE, Nutritionix, natural-language understanding, and sentiment analysis, reporting 1,007 chatbot responses to 892 user input messages. While the study subsequently classified 601 responses as accurate and 189 as inaccurate, the published percentages (67.38% and 21.19%, respectively) correspond to a denominator of 892 rather than the stated denominator of 1,007, indicating an internal inconsistency that limits the report’s reliability. Consequently, the study serves primarily to inform interface design rather than provide clinical evidence, given its simulated setting, very small convenience sample, and absence of objective outcomes (27).

Advancing beyond rule-based systems, a hybrid generative nutrition system combined variational autoencoder/gated recurrent unit models, guideline-informed loss functions, optimiser-based portion adjustment and GPT-3.5-assisted meal-database expansion. Evaluation used 3,000 simulated profiles to generate 84,000 daily plans and 1,000 profiles sampled from two public datasets to generate 7,000 plans. Generated plans generally aligned with energy and nutrient targets, but the study lacked prospective patient testing, adherence assessment, clinical outcomes and safety evaluation. Similar limitations were noted in exercise planning, where GPT-4 could generate structured 30-day exercise plans, yet expert evaluation identified inadequate integration of contraindications, health status and real-time physiological feedback (28, 29).

In contrast to these simulated or technical assessments, earlier observational evidence from the fully automated Lark coach showed a mean weight change of −2.38% among 70 selected engaged users over approximately 15 weeks, but the absence of a comparator, reliance on user-entered data and selection of engaged users preclude causal inference (30). Current chatbot and generative-AI evidence therefore supports feasibility, acceptability and communication more strongly than sustained clinical weight loss. Except for a limited number of structured or prescription digital programmes, most systems reviewed here remain research prototypes or small-study interventions and have not been widely adopted in routine obesity care. Their present role is limited to clinician-reviewed education, reminders and draft advice rather than autonomous treatment (**Table 3**).

**Table 3:** Conversational agents and generative AI in weight management and bariatric surgery.

Study	System and task	Design and data	Key findings	Main limitations	Current interpretation
Stein and Brooks (2017) (30)	Lark fully automated text-based AI health coach for weight loss and diabetes-prevention behaviours.	Longitudinal observational study of 70 engaged adults with overweight or obesity; mean use 15 weeks.	Mean weight change −2.38% (SE 0.69%); users averaged 103 sessions; proportion of healthy logged meals increased by 31%.	No comparator; self-reported weight and diet; highly selected engaged-user sample; unmeasured confounding and no causal inference.	Early real-world feasibility and engagement evidence; controlled trials are needed to establish efficacy.
Maher et al. (2020) (26)	Paola, an IBM Watson-based virtual coach supporting physical activity and a Mediterranean-style diet.	Twelve-week single-arm proof-of-concept study; 31 adults aged 45–75 years.	Physical activity +109.8 min/week; Mediterranean diet score +5.7; weight −1.3 kg; waist −2.1 cm. Twelve-week retention was 90%.	No randomised comparator; small sample; smartphone/technical barriers. Approximately 25% did not complete the first independent weekly check-in, which is not equivalent to overall attrition.	Supports feasibility and acceptability; clinical effectiveness remains uncertain.
Rahmanti et al. (2022) (27)	SlimMe chatbot using Dialogflow, LINE, Nutritionix, natural-language understanding and sentiment analysis.	Seven-day simulation with 10 international female students; five had nutrition/dietetics backgrounds; 1,007 bot responses.	The study reported 1,007 chatbot responses to 892 user messages; however, the response-category counts and percentages were internally inconsistent with the stated denominator.	Very small convenience sample, simulated setting, normal mean BMI, no comparator and no objective clinical outcome.	Useful for interface-error characterisation, not evidence of weight-loss effectiveness or clinical safety.
Papastratis et al. (2024) (28)	Hybrid nutrition-recommendation system using VAE/GRU models, guideline-informed loss functions, optimiser-based portion adjustment and GPT-3.5-assisted meal-database expansion.	Evaluation with 3,000 simulated profiles and 84,000 daily plans; additional testing used 1,000 profiles sampled from two public datasets and 7,000 daily plans.	Generated diverse plans that generally aligned with energy and nutrient targets; optimisation and guideline constraints were integral to alignment.	Simulation/public datasets; no prospective patients, clinical outcomes, adherence assessment or safety evaluation.	Technical/hypothesis-generating evidence; should not be labelled simply as a ChatGPT intervention.
Dergaa et al. (2024) (29)	GPT-4 generation of personalised 30-day exercise programmes.	Five hypothetical participant profiles; single-prompt generation; expert qualitative evaluation.	GPT-4 produced structured programmes, but experts identified incomplete integration of contraindications, individual health status, goals and real-time physiological feedback.	Only five simulated profiles; no validated rating scale, repeated-run analysis, patient testing or clinical outcomes; model performance is version- and prompt-dependent.	Suitable for clinician-reviewed drafting or hypothesis generation, not autonomous exercise prescription.
Lee et al. (2024) (8)	ChatGPT-4, Google Bard and Microsoft Bing answering guideline-derived bariatric-surgery questions.	Remote evaluation; randomised, blinded duplicate review by accredited bariatric surgeons; appropriateness rated on a 5-point scale and readability assessed with FRE.	ChatGPT-4: 4.46/5, 85.7% appropriate, FRE 21.68. Bard: 3.89, 74.3%, FRE 42.89. Bing: 3.11, 25.7%, FRE 14.64.	One prompt set and historical model versions; appropriateness is not diagnostic accuracy; low readability and no real-patient decisions or clinical outcomes.	ChatGPT-4 performed best in this comparison but still required clinician oversight and patient-language simplification.
Lopez-Gonzalez et al. (2025) (13)	ChatGPT-4 recommendation of bariatric procedure using individual patient data.	Single-centre retrospective study of 161 real patients; AI recommendations compared with the hospital algorithm and actual decisions.	Recommended RYGB in 56.5%, SG in 37.9%, endoscopic techniques in 4.3% and DS in 1.2%; concordance with actual clinical decisions was 34.16%; disagreement was 65.84%.	Single centre; incomplete representation of multidisciplinary context, patient preference and local pathways; no prospective decision-impact evaluation.	Insufficient concordance for autonomous procedure selection.
Sanchez-Cordero et al. (2025) (59)	ChatGPT procedure recommendations before and after contextual exposure to 412 open-access bariatric articles.	Single-centre retrospective study of 283 patients; recommendations compared with the institutional technique-selection protocol.	Baseline concordance/disagreement was 20.0%/80.0%; after contextual exposure to 412 articles, concordance/disagreement was 25.8%/74.2%.	Context conditioning was not parameter retraining; single centre and model session; limited reproducibility and no prospective clinical evaluation.	Additional literature altered output distributions but did not reproduce individualised clinical judgement.
Kahlon et al. (2025) (60)	ChatGPT-4 vs ASMBS-accredited surgeons for medical or surgical management of five bariatric scenarios.	Five clinical vignettes; two independent ChatGPT runs; comparison with surgeons and pre-2022 ASMBS guidance selected because of the model’s 2021 knowledge cutoff.	Guideline concordance: surgeons 100%, ChatGPT 60%. Agreement with surgeon: κ=0.37 (95% CI −0.14 to 0.89) and κ=0.28 (−0.25 to 0.82); between ChatGPT runs κ=0.44 (−0.20 to 1.00).	Only five vignettes, very wide CIs, older guideline benchmark and no patient outcomes. Agreement was weaker for complex postoperative/metabolic cases.	Demonstrates instability and limited contextual judgement; not suitable for unsupervised high-risk decisions.

AI, artificial intelligence; ASMBS, American Society for Metabolic and Bariatric Surgery; CI, confidence interval; DS, duodenal switch; FRE, Flesch Reading Ease; GPT, generative pretrained transformer; GRU, gated recurrent unit; κ, Cohen’s kappa; RYGB, Roux-en-Y gastric bypass; SE, standard error; SG, sleeve gastrectomy; VAE, variational autoencoder. Commercial-model results are time-, version- and prompt-dependent and should not be interpreted as current universal performance.

### 3.3 Synthesis: what is effective, what is AI-specific, and what remains uncertain

The active ingredients of successful digital programmes are behavioural and organisational as well as algorithmic: education, self-monitoring, goal setting, timely feedback, connected scales or wearables, remote follow-up and human supervision. AI may improve automation, personalisation and scalability, but dismantling studies capable of separating these contributions are rare. This interpretation is consistent with the systematic review by Hallock et al., which included 21 studies—eight randomised controlled trials and 13 observational studies—of AI-enabled behavioural coaching platforms. Although the review reported promising weight and cardiometabolic outcomes and retention rates of 57–92%, particularly in hybrid models combining AI with human coaching, heterogeneity, short study duration, algorithmic opacity, limited cultural tailoring and unequal technological access restricted conclusions regarding durable effectiveness and equitable implementation (9).

Evidence quality is greatest when outcomes are objective and follow-up is longer. Oviva used supervised calibrated weights, Lee et al. reported HbA1c and connected-device data, and AI-DPP used a 12-month randomised noninferiority design. AI-DPP provides strong comparative evidence for automated delivery with higher observed programme initiation, but it demonstrated noninferior, not superior, clinical outcomes relative to human coaching. In contrast, the 1-week Chew study and small chatbot studies primarily provide behavioural or usability signals. Generalisability is further limited by country-specific reimbursement systems, digital literacy, selected active-user populations and possible developer involvement (5, 21, 22, 24).

## 4 AI in anti-obesity pharmacotherapy and drug development

GLP-1 receptor agonists and the dual GIP/GLP-1 receptor agonist tirzepatide have produced substantial and clinically meaningful weight loss in randomised trials. Because obesity is a chronic and relapsing disease, discontinuation of weight-management medication is commonly followed by weight regain and attenuation of cardiometabolic benefits. Such regain should be interpreted in the context of treatment withdrawal and the underlying chronic disease rather than as evidence that the medication was ineffective during treatment. Important challenges in long-term pharmacotherapy include heterogeneity in response, gastrointestinal adverse effects, tolerability, adherence, affordability, access and continuity of care (3, 31–33). Within this heterogeneous evidence base, AI applications should be evaluated according to their intended use rather than grouped broadly as AI-guided pharmacotherapy. Three domains can be distinguished: AI-assisted drug discovery and molecular optimisation; NLP-based analysis of patient experience and pharmacovigilance signals; and responder stratification or precision pharmacotherapy. These domains differ substantially in evidence maturity. Drug-discovery studies remain predominantly preclinical or early translational; text-mining studies primarily support patient-experience characterisation, signal detection and hypothesis generation rather than causal inference or quantitative safety estimation; and responder-stratification studies provide biological and candidate clinical evidence, but among the eligible evidence screened and included in this review, we identified no externally validated, prospectively implemented AI prescribing system that demonstrated improved patient outcomes.

### 4.1 Drug discovery and molecular optimisation: preclinical and translational evidence, not clinical effectiveness

AI-assisted drug discovery may improve target identification, virtual screening, candidate prioritisation, lead optimisation and peptide design, but its clinical value cannot be established until pharmacokinetic, toxicological and human efficacy studies are completed (6). Cho et al. combined Bayesian classification with three-dimensional pharmacophore modelling to identify a new anti-obesity chemotype from natural piperidine compounds. Shin et al. used sequential quantitative structure–activity relationship (QSAR) models to prioritize glucocorticoid-receptor-antagonist phytochemicals. Both studies illustrate more efficient screening of complex chemical spaces, but remain primarily *in vitro* or preclinical (34, 35). For peptide and multi-target development, a deep multitask neural network designed GCGR/GLP-1R dual-agonist sequences with enhanced biological potency, while machine-learning-guided QSAR screening of 2688 secretin-scaffold peptides generated GLP-1R agonist candidates with improved drug-like properties. These studies show translational promise but not clinical effectiveness in obesity (36, 37). Accordingly, AI drug discovery should be described as preclinical or early translational evidence. Key uncertainties include overfitting, chemical-space generalisability, toxicity, pharmacokinetics and translation from experimental systems to humans.

### 4.2 Patient experience and pharmacovigilance: signal detection, not causal inference or safety quantification

AI and NLP can extract perceived effectiveness, adverse effects, treatment discontinuation, cost and access barriers from patient narratives and social media, providing data that are valuable for early signal detection and hypothesis generation. However, these data usually lack verified diagnoses, doses, treatment duration, concomitant medication and longitudinal outcomes; consequently, they cannot establish causality or adverse-event rates (38, 39).

For example, Bhagavathula analysed 772 Drugs.com reviews of semaglutide for weight loss using Bidirectional Encoder Representations from Transformers (BERT) sentiment analysis, topic modelling and clustering. The analysis identified heterogeneity in perceived effectiveness, tolerability, adherence and access, but interpretation is limited by selective posting, self-report, absent clinical verification and uncertain representativeness (38). In a larger-scale analysis, Somani et al. analysed 391,461 GLP-1 receptor agonist-related Reddit discussions from 116,216 authors and identified 168 topics in 33 groups. The system captured known adverse effects and potential signals involving menstrual changes, fertility, anxiety, depression and myalgia. However, events were unverified, Reddit users were not representative of all patients, and the LLM labelling system was not specifically validated for pharmacovigilance (39). Similarly, Javaid et al. classified 14,390 Reddit posts from 8412 authors into 30 topics covering weight loss, HbA1c, cost, insurance denial, administration, adverse effects and off-label use. General-purpose sentiment models sometimes misclassified medical terms such as ‘weight loss’ and ‘side effects’ as negative. Analyses of semaglutide discussions on X face similar limitations involving platform representativeness, authenticity and algorithmic misclassification (40, 41).

The appropriate role of these methods is to complement formal pharmacovigilance and prioritize signals for verification using electronic health records, prescription data, claims, adverse-event reporting and prospective patient-reported outcomes.

### 4.3 Responder stratification and precision pharmacotherapy: candidate evidence without validated AI-guided prescribing

Responder stratification is clinically attractive because obesity treatment response varies with appetite, satiation, gastric emptying, energy expenditure, eating behaviour, metabolic status, genetics, microbiome, tolerability, affordability and adherence. Current evidence includes biological rationale, phenotype-guided treatment and candidate predictors, including a machine-learning-assisted genetic risk score. However, among the eligible studies screened, we identified no large, multicentre, cross-ancestry, externally validated and interpretable AI prescribing system, or randomised trial demonstrating benefit from AI-guided medication selection (42, 43). Dynamic phenotyping has classified obesity according to satiation, postprandial satiety, energy expenditure and emotional eating. In a pragmatic clinic study, phenotype-guided medication selection was associated with greater 12-month weight loss than usual care. This supports phenotype-based treatment, but not prospective validation of an AI prescribing system (44, 45).

Gastric emptying, satiation and early weight change are plausible model inputs. A randomised liraglutide pilot linked treatment with changes in weight, satiation and gastric function, while early response at 16 weeks predicted greater weight loss at 56 weeks. These studies identify candidate features for future AI models rather than validating existing AI-guided prescribing systems (46, 47).

Genetic, microbiome, metabolomic and lipidomic data may add biological information, but sample size, batch effects, cross-platform comparability and ancestry portability remain major barriers (48–51).Polygenic scores often perform less well outside European-ancestry populations, making diverse multicentre datasets and direct cross-ancestry validation essential (52).

Recent work integrating genetic, physiological and behavioural features used a machine-learning-assisted genetic risk score and reported associations with differential treatment response. The calories-to-satiation genetic risk score (CTS_GRS) was developed and validated as a predictor of satiation, and treatment-response associations were examined retrospectively within two relatively small randomised-trial cohorts (n = 50 for phentermine–topiramate and n = 110 for liraglutide). Treatment allocation was not guided by the score; therefore, the analysis supports predictive enrichment rather than clinical utility of AI-guided prescribing. Independent external validation, cross-ancestry transportability, prospective workflow implementation and demonstrated clinical benefit remain lacking (53). These findings provide biological plausibility and candidate predictive information but do not establish a validated basis for AI-guided prescribing. Prospective studies must test whether AI-guided drug selection or dose adjustment outperforms clinician-led care for weight loss, tolerability, adherence, cost and equity.

## 5 AI in metabolic and bariatric surgery

AI applications in metabolic and bariatric surgery (MBS) include preoperative counselling and risk assessment, operative-time prediction, anaesthetic monitoring, surgical video analysis, postoperative complication and readmission prediction, weight-trajectory modelling and digital follow-up. Reviews consistently show expanding technical work but limited prospective clinical-impact evidence (12, 54, 55). Surgical AI should be judged not only by accuracy or AUROC, but also by calibration, decision-curve analysis, external validation, actionable thresholds, workflow integration, alert burden, subgroup fairness and prospective impact. A clinically useful model must specify what action follows a high-risk prediction and whether that action improves outcomes or resource use (14, 18, 19).

### 5.1 Preoperative counselling and risk assessment

Preoperative applications include education, procedure recommendations, difficult-airway prediction and operative-time estimation. Their clinical relevance should be judged by guideline concordance, expert review and whether outputs are linked to prespecified workflow actions rather than by the presence of a model-generated recommendation alone.

#### 5.1.1 Large language models for bariatric counselling and clinical decision support

LLMs have been evaluated for guideline-based questions, patient education and procedure recommendations. Their appraisal should include appropriateness, readability, reproducibility, citation reliability, hallucination risk and concordance with real clinical decisions rather than linguistic fluency alone. Two recent obesity-specific reviews support this cautious interpretation. Suenghataiphorn et al. identified 33 studies spanning personalised nutrition, education, guideline-directed treatment, weight-loss strategies, anti-obesity medication information and motivational interviewing. Performance varied substantially, with inconsistent recommendations, factual inaccuracies, difficulty managing complex clinical scenarios and potentially biased outputs, supporting continued clinician oversight and real-world validation (10). Motevalli et al. included 37 studies, comprising 29 original and eight review-based studies, but rated only ten studies (27%) as high confidence. Although ChatGPT showed promising performance in several lifestyle, nutrition and bariatric-surgery evaluations, comparisons with specialised clinical applications remained scarce, and concerns regarding reliability, bias, cultural sensitivity, transparency, excessive dependence, and ethical and legal accountability continued to limit clinical deployment (11).

Reproducible studies should report model name and version, access date, prompts, external knowledge sources, repeated runs, output variability, rater expertise, inter-rater agreement, reference standards and the potential consequences of errors. Commercial model updates make these details essential (56, 57).

In guideline-derived questions, ChatGPT-4 achieved a mean appropriateness score of 4.46/5 and 85.7% appropriate responses, compared with 3.89 and 74.3% for Bard and 3.11 and 25.7% for Bing. However, ChatGPT-4’s mean Flesch Reading Ease score was 21.68, indicating college-level complexity, and 14.3% of answers remained inappropriate. Thus, relatively strong question-answering performance did not ensure patient readability or unsupervised safety (8). BariatricSurgeryGPT was a technical proof-of-concept in which GPT-2 was fine-tuned on 8,764 bariatric-surgery abstracts and evaluated on 20 questions using automated text-similarity metrics, including BLEU, METEOR and ROUGE. The study did not use an expert clinical truth standard, real treatment decisions or patient outcomes; domain adaptation therefore did not establish reliable procedure selection, complication management or clinical decision support (58). Lopez-Gonzalez et al. tested ChatGPT-4 using data from 161 real patients. The model recommended Roux-en-Y gastric bypass in 56.5%, sleeve gastrectomy in 37.9%, endoscopic techniques in 4.3% and duodenal switch in 1.2%. Concordance with actual clinical decisions was 34.16%, corresponding to disagreement in 65.84% of cases (13). Sanchez-Cordero et al. evaluated 283 patients. Before contextual exposure, concordance was 20.0% and disagreement was 80.0% (κ = 0.003; P = 0.96). After contextual exposure to 412 open-access articles, concordance was 25.8%, while disagreement remained 74.2% (κ = 0.068; P = 0.29) (59). Across only five clinical vignettes, Kahlon et al. found stronger agreement in simple cases but important disagreement in complex postoperative or metabolic cases. Guideline concordance was 100% for surgeons and 60% for ChatGPT. Agreement with surgeons was fair but imprecise (κ = 0.37, 95% *CI* −0.14 to 0.89; and κ = 0.28, 95% *CI* −0.25 to 0.82), and consistency across two model runs was moderate with a wide confidence interval (κ = 0.44, 95% *CI* −0.20 to 1.00). The very small vignette set and wide confidence intervals preclude broad generalisation (60). These data define a clear boundary: LLMs may assist education and drafting, but should not independently select bariatric procedures or manage complex postoperative problems.

Real MBS decisions integrate BMI, diabetes, reflux, hiatal hernia, prior surgery, nutritional risk, patient preference, anaesthetic risk, local pathways, insurance and team expertise. General-purpose LLMs rarely have complete verified data or reliable access to local context, limiting individualized risk-benefit judgement (4). Hallucinations, incorrect citations, overconfident language and unstable outputs remain important safety risks, especially for high-risk treatment, triage and perioperative decisions (57, 61, 62). Retrieval-augmented generation (RAG) can ground answers in guidelines or institutional knowledge and may improve factual consistency and traceability, but performance still depends on source quality, retrieval, segmentation, updating and human review. Medical studies show improvement in some question-answering and preoperative-assessment tasks, not elimination of error (63–67).

For now, reasonable LLM uses include clinician-reviewed education, consultation preparation, plain-language explanation and documentation support. High-risk decision support requires prospective validation, auditable evidence sources and a clinician-in-the-loop workflow.

#### 5.1.2 Comprehensive preoperative risk assessment

Preoperative prediction models can integrate comorbidity, laboratory, anaesthetic and procedure data, but a risk score is useful only when externally validated, calibrated and linked to a defined clinical response. A machine-learning model for difficult intubation in patients with obesity showed potential for identifying airway risk. Independent validation and threshold testing are still needed before predictions can reliably trigger senior anaesthetist involvement, video-laryngoscope preparation or enhanced postoperative monitoring (68). Machine-learning prediction of operative time may improve scheduling and resource use. Relevant evaluation should emphasize absolute prediction error, performance across procedures and surgeons, and whether implementation reduces delays, cancellations or overtime rather than merely reporting model fit (69).

### 5.2 Intraoperative monitoring and computer vision

Intraoperative AI in metabolic and bariatric surgery has been evaluated primarily through computer-vision analysis of operative video. Evaluation should report phase-specific performance, real-time error patterns and whether outputs improve training, workflow or safety rather than relying on overall accuracy alone. Hashimoto et al. reported a mean test accuracy of 82% (SD 4%) and a maximum accuracy of 85.6% for recognising seven operative steps in 88 laparoscopic sleeve-gastrectomy videos (7). A subsequent retrospective single-centre implementation study of 49 consecutive sleeve-gastrectomy videos demonstrated the feasibility of automated workflow analysis and reported agreement with surgeon assessment for four of five predefined safety milestones (70). Potential applications include workflow quantification, surgical training, case review and quality assessment.

Clinical translation requires phase-specific performance, centre- and surgeon-level external testing, assessment of video quality and data leakage, and evidence that outputs improve teaching, safety steps or postoperative outcomes. Current evidence does not support autonomous surgical navigation.

### 5.3 Early postoperative complication and readmission prediction

Postoperative models have targeted serious complications, gastrointestinal leak, venous thromboembolism (VTE) and 30-day readmission. Because these outcomes are often uncommon, high accuracy can coexist with poor sensitivity or positive predictive value. Calibration, net benefit, alert burden and the consequences of missed or false-positive cases are therefore essential (71–73).

Using Scandinavian registry data (37,811 training and 6250 test patients; serious-complication incidence approximately 3%), Cao et al. compared 29 algorithms. Although accuracy and specificity commonly exceeded 90%, sensitivity was only about 36.4%-41.7% and most AUROCs were close to 0.5. The models were exploratory and lacked adequate calibration, decision-curve analysis and independent geographical validation (71). A subsequent deep-learning analysis of the same registry found very high specificity but near-zero sensitivity in the imbalanced dataset. SMOTE improved training performance, but test performance remained unstable, illustrating that oversampling does not guarantee generalisability for rare outcomes (72). Nudel et al. analysed 436,807 Metabolic and Bariatric Surgery Accreditation and Quality Improvement Program (MBSAQIP) patients (leak 0.70%; venous thromboembolism [VTE] 0.46%). For leak, AUROC was 0.75 for an artificial neural network, 0.70 for XGBoost and 0.63 for logistic regression. For VTE, AUROC was 0.65 for the artificial neural network, 0.67 for XGBoost and 0.64 for logistic regression. These results suggest moderate discrimination for leak and limited discrimination for VTE, without proof that implementation reduces either event (73). The original BariClot study provided an early bariatric-specific framework for estimating 30-day VTE risk. Using MBSAQIP data from 791 centres, Dang et al. analysed 274,221 patients undergoing primary laparoscopic Roux-en-Y gastric bypass or sleeve gastrectomy. VTE occurred in 1,106 patients (0.4%), including 452 pulmonary embolic events (0.2%), and 43 patients (0.02%) died from VTE, which was the most frequently identified cause of 30-day mortality. A forward-selection logistic-regression model incorporating previous VTE, operative time, race and functional status was developed in a randomly generated derivation cohort and evaluated in a separate held-out cohort from the same registry. BariClot classified predicted risk as low (<0.3%), medium (0.3–1%), high (1–2%) or very high (>2%), providing a potentially simple framework for identifying patients who might be considered for intensified surveillance or extended thromboprophylaxis. However, this represented split-sample internal validation rather than independent external validation, and the study did not establish that using the score to guide prophylaxis reduced thromboembolic events or improved net clinical benefit. The inclusion of race as a predictor also requires careful assessment of transportability, subgroup performance and fairness before clinical deployment (74). Butler et al. developed readmission models in 863,348 MBSAQIP patients (readmission rate 4.52%). XGBoost and random forest each achieved an AUROC of 0.785, neural networks 0.754 and logistic regression 0.620. At 70% specificity, XGBoost sensitivity was 73.81% versus 52.94% for logistic regression. Better discrimination did not establish that alerts improve discharge planning or reduce readmissions (75).

These models should currently be viewed as adjuncts for risk stratification. Progress requires independent external validation, calibration, decision-curve analysis, actionable thresholds and prospective testing of linked interventions such as extended observation, earlier imaging or intensified follow-up.

### 5.4 Long-term follow-up and weight-trajectory prediction

Long-term postoperative management requires monitoring of weight trajectories, nutritional status, adherence, weight regain, metabolic outcomes and patient-reported symptoms. AI and digital platforms can combine mobile, wearable and clinical data, but prediction or alert generation should not be equated with improved long-term outcomes.

#### 5.4.1 Long-term weight-trajectory prediction

SOPHIA was developed using multinational retrospective cohorts to predict 5-year BMI trajectories after bariatric surgery. Although its international external testing and interpretable continuous outputs are strengths, the development study did not demonstrate that clinical use improves individual counselling, procedure selection, weight maintenance, resource allocation or patient satisfaction (76). In an independent comparison of 178 patients, the proportion whose observed weight remained within the predicted interquartile range declined from 50.7% at 12 months to 43.2% at 24 months and 38.8% at 60 months; consequently, 61.2% of patients were outside the predicted range by five years. At 60 months, the mean actual-versus-predicted difference was only 0.6 kg, but the corresponding SD was 16.7 kg, and the median absolute deviation was 12.9 kg (95% CI 11.3–14.4), indicating substantial individual-level error (77). The small mean difference may appear reassuring because positive and negative prediction errors cancel at the group level, but it should not be interpreted as stable prediction for an individual patient. Current performance therefore does not establish that SOPHIA is sufficiently reliable as a stand-alone tool for individual counselling or procedure selection; any clinical use should be adjunctive, explicitly communicate prediction uncertainty and be supported by further prospective evaluation. Earlier studies used several computational approaches to predict postoperative weight outcomes, although they addressed different prediction tasks. Piaggi et al. studied 172 women undergoing laparoscopic adjustable gastric banding and used preoperative age, body mass index and Minnesota Multiphasic Personality Inventory-2 psychometric measures to predict excess weight loss (EWL) at 24 months. A multilayer-perceptron model based on age and three selected psychological scales explained 36.5% of the variation in EWL, compared with 10% for multiple linear regression, and achieved a classification accuracy of approximately 69.8% at the selected threshold. Repeated three-fold cross-validation provided some assessment of internal performance, but the model was developed in a small, women-only cohort undergoing a procedure that is now less representative of contemporary bariatric practice. It was not evaluated in an independent external cohort, and calibration and clinical utility were not reported (78).

Dimeglio et al. explicitly modelled longitudinal weight trajectories after gastric bypass. Hierarchical cluster analysis of 795 patients with at least 2 years of follow-up generated 14 representative trajectories, which were grouped by clinical assessment into four families: continuous loss or subsequent stabilisation; loss followed by regain and renewed loss; loss followed by regain; and initial stagnation followed by later loss. The model was evaluated in a second group of 381 patients with 5-year follow-up. Weight measurements during the first 6 postoperative months correctly classified 98% of patients who ultimately achieved EWL >50%, but performance exceeded only 58% among those with EWL of 25–50%. Interpretation is limited by the highly imbalanced evaluation cohort, in which 87.4% achieved EWL >50%, only 12.6% achieved EWL of 25–50%, and no patients met the category of weight-loss failure. Moreover, the evaluation was conducted within a single-centre population and did not establish that trajectory-based alerts altered subsequent management or prevented weight regain (79).

Collectively, these studies demonstrate technical feasibility but do not establish clinical effectiveness. The most plausible role of long-term prediction is as a dynamic monitoring and early-warning tool rather than as a preoperative gatekeeping mechanism. Contemporary models should incorporate repeated postoperative weight measurements together with nutritional intake, physical activity, clinic attendance, psychological or behavioural factors and use of adjunctive anti-obesity medication. Prospective multicentre studies should assess temporal and external validation, calibration across follow-up intervals, subgroup performance, missing-data robustness and decision-curve utility. Most importantly, clinical-impact studies must determine whether model-triggered dietitian review, behavioural support, anatomical assessment or pharmacotherapy improves total weight loss, prevents clinically important weight regain and reduces loss to follow-up without generating excessive alerts, unnecessary treatment or inequitable care.

#### 5.4.2 Digital and AI-assisted continuous postoperative monitoring

Dynamic postoperative systems can collect weight, intake, hydration, symptoms, activity and patient-reported outcomes. Evaluation should include response rates, false alerts, missed events, escalation pathways, staff workload, digital literacy, equity and cost-effectiveness rather than model performance alone.

A three-tiered mobile follow-up system achieved 67.5% response adherence and 94% satisfaction; red+ alerts were associated with complications, emergency visits and readmission. The non-randomised study demonstrated feasibility and risk signalling, not that alerts reduced these outcomes (80). A subsequent model incorporating dynamic application alerts achieved an AUROC of 0.715 and accuracy of 77.4%, outperforming static preoperative variables. Sensitivity, specificity, calibration, external validation, decision curves and prospective impact remain incompletely established (81).

Digital and AI-assisted follow-up is therefore a promising adjunct for prioritising review, but it requires workflow trials showing reduced delay, complications, emergency visits, readmission or workload without unacceptable alert fatigue or inequity.

### 5.5 Clinical translation of bariatric surgical AI

Overall, bariatric AI has progressed furthest in technical feasibility and retrospective risk stratification, while evidence of clinical impact remains absent. Of the eight representative studies summarised in **Table 4**, six were retrospective (75%). The two prospective reports each involved a small, single-centre cohort of 104 patients. One evaluated a predefined, clinician-designed three-tiered alert system and did not use a machine-learning prediction model; its findings demonstrated feasibility, acceptability and associations between alert profiles and adverse events, but not that the alert system reduced those events (80). The other prospectively collected digital follow-up data to develop and internally validate a machine-learning complication model, but the model was not prospectively implemented to guide clinical decisions or tested for effects on complications, emergency-department visits, readmissions or other patient outcomes (81). Consequently, none of the eight representative studies demonstrated improved patient outcomes resulting from prospective AI-guided implementation. **Table 4** therefore primarily documents technical feasibility, retrospective model performance and digital-monitoring feasibility rather than clinical effectiveness. An international expert consensus involving 68 specialists from 35 countries reached consensus on 28 statements and identified education and training, decision support and planning, ethical guidance, informed consent, transparency and avoidance of overreliance as priorities for responsible adoption (82). The appropriate current role of these technologies is clinician-supervised risk identification, communication and follow-up prioritisation. Transition to routine care requires externally validated models, explicit action thresholds and prospective implementation studies demonstrating clinical net benefit, safety, fairness and sustainable resource use.

**Table 4:** Representative digital and AI applications across metabolic and bariatric surgery beyond large language models.

Study	Application	Design and data	Digital/AI approach	Key performance or findings	Validation, limitations and current interpretation
Hashimoto et al. (2019) (7)	Intraoperative workflow recognition	Retrospective analysis of 88 laparoscopic sleeve-gastrectomy videos from one academic institution; surgeon-annotated operative steps.	Deep neural networks for seven-step operative-phase recognition; 70% training and 30% testing split.	Mean test accuracy 82% (SD 4%); maximum accuracy 85.6%. Human annotator concordance correlation coefficient 0.862.	Single-centre internal test, no patient-outcome or workflow-impact evaluation and potential sensitivity to video quality and local technique. Supports technical feasibility, not autonomous navigation.
Cao et al. (2019) (71)	Severe 30-day complication prediction	Swedish registry; 37,811 patients for training (2010–2014) and 6,250 for temporal testing (2015); event incidence approximately 3%.	Twenty-nine supervised ML approaches, including ensemble and SMOTE-based models.	Accuracy and specificity commonly exceeded 90%, but best test sensitivities were only 41.7% and 36.4%; most test AUROCs were near 0.50. Deep NN test AUROC was 0.56.	Rare-event imbalance made accuracy misleading. Temporal rather than geographical external testing; inadequate sensitivity, calibration and clinical-action evaluation. Not ready for clinical deployment.
Nudel et al. (2021) (73)	Leak and VTE prediction	Retrospective MBSAQIP cohort of 436,807 operations; leak incidence 0.70% and VTE incidence 0.46%; random train/validation/test split.	Artificial neural network and XGBoost compared with logistic regression using preoperative variables.	Leak AUROC: 0.75 (ANN), 0.70 (XGBoost), 0.63 (logistic regression). VTE AUROC: 0.65, 0.67 and 0.64, respectively.	Internal validation only; moderate discrimination for leak and limited discrimination for VTE. No calibration, decision-curve or implementation evidence demonstrating fewer complications.
Butler et al. (2023) (75)	Thirty-day readmission prediction	Retrospective MBSAQIP analysis of 863,348 operations; 39,068 readmissions (4.52%).	XGBoost, random forest and neural networks compared with logistic regression; models included pre-, intra- and postoperative variables.	AUROC: 0.785 for XGBoost and random forest, 0.754 for neural networks and 0.620 for logistic regression. At 70% specificity, sensitivity was 73.81% vs 52.94%.	Performance depended partly on postoperative information already available before discharge. Internal validation only; external validation and trials of linked discharge/follow-up interventions are required.
Saux et al. (2023) (76)	Five-year weight-trajectory prediction	Multinational retrospective SOPHIA study; 10,231 patients from 12 centres in 10 countries after gastric bypass, sleeve gastrectomy or gastric banding.	LASSO variable selection and interpretable classification-and-regression trees using seven baseline variables.	Across external testing cohorts at 5 years: mean BMI MAD 2.8 kg/m^2^ (95% CI 2.6–3.0), RMSE 4.7 kg/m^2^ (4.4–5.0) and mean predicted–observed difference −0.3 kg/m^2^ (SD 4.7).	International external testing is a strength, but the study was retrospective. Clinical use did not demonstrate improved counselling, procedure selection, weight maintenance or resource allocation.
Sankar et al. (2025) (77)	Independent evaluation of SOPHIA	Retrospective external comparison in 178 UK patients with 5-year follow-up; 83.1% underwent gastric bypass.	SOPHIA-predicted trajectories compared with measured annual weights.	Actual weight was within the predicted IQR in 50.7%, 43.2% and 38.8% at 12, 24 and 60 months. At 60 months, mean actual–predicted difference was 0.6 kg (SD 16.7) and MAD was 12.9 kg (95% CI 11.3–14.4).	Precision declined over time and varied by procedure/subgroup; the cohort was small and predominantly gastric bypass. A small mean difference concealed substantial individual error.
Farinella et al. (2025a) (80)	Remote digital postoperative follow-up and alert feasibility	Prospective, single-centre, non-randomised cohort of 104 patients using a 60-day mobile follow-up system after bariatric surgery.	Predefined, clinician-designed three-tiered alert system (orange, red and red+); no machine-learning prediction model was evaluated.	1,119 alerts; response adherence 67.5% and satisfaction 94%. Red+ alert profiles were associated with approximately threefold higher morbidity, emergency department visits and readmission.	Small observational cohort with self-reported responses. Alert-outcome associations do not establish that the system reduced adverse outcomes or resource use. Supports feasibility and acceptability only.
Farinella et al. (2025b) (81)	Dynamic prediction of postoperative complications	Prospective, single-centre cohort of 104 patients; model development used a 72/32 training/validation split with fivefold cross-validation.	Supervised logistic regression using six selected dynamic features derived from patient-reported symptoms, responses and alert counts; compared with a preoperative linear-discriminant model.	Postoperative model: accuracy 77.4%, F1 46.2% and AUROC 0.715. Preoperative model: accuracy 75.0%, F1 43.0% and AUROC 0.647.	Small, single-centre dataset with class imbalance and internal validation only; no independent external validation, calibration, decision-curve analysis or prospective clinical-impact evaluation. Supports technical feasibility, not clinical effectiveness.

AI, artificial intelligence; ANN, artificial neural network; AUROC, area under the receiver operating characteristic curve; BMI, body mass index; CI, confidence interval; F1, harmonic mean of precision and recall; IQR, interquartile range; LASSO, least absolute shrinkage and selection operator; MAD, median absolute deviation; MBSAQIP, Metabolic and Bariatric Surgery Accreditation and Quality Improvement Program; ML, machine learning; NN, neural network; RMSE, root mean squared error; SMOTE, synthetic minority oversampling technique; VTE, venous thromboembolism. Metrics are not directly comparable across studies because outcomes, datasets and validation designs differ.

Postoperative prediction benefits from large registries, yet rare-event imbalance, limited calibration, absent decision-curve analysis and lack of independent external validation restrict clinical utility. Long-term trajectory and digital follow-up tools are relevant to real workflows but retain substantial individual error and have not demonstrated outcome improvement. The appropriate current role is clinician-supervised risk identification, communication and follow-up prioritisation. Transition to routine care requires externally validated models, explicit action thresholds and prospective implementation studies demonstrating clinical net benefit, safety, fairness and sustainable resource use. Representative non-LLM digital and AI applications in metabolic and bariatric surgery are summarised in **Table 4**.

## 6 Discussion and critical appraisal

The evidence should be interpreted according to the type and level of support provided rather than by a binary question of whether AI “works”. Across obesity care, studies variously demonstrate technical feasibility, discrimination, acceptability, decision-support potential or patient-level benefit; these outcomes are not interchangeable. Direct patient-level evidence for explicitly AI-enabled obesity interventions remains limited. The strongest patient-level weight or metabolic outcome evidence derives from broader multicomponent digital, automated or hybrid-care programmes; these studies support the complete delivery model rather than a separable AI effect. Chatbots and generative nutrition or exercise tools remain early-stage. Small samples, short follow-up, self-report and simulation designs support usability and communication, but not durable clinical efficacy or unsupervised treatment. Pharmacotherapy-related AI spans preclinical discovery, hypothesis-generating pharmacovigilance and biologically plausible precision treatment. Within the evidence included in this review, we identified no evidence sufficient to support routine AI-guided medication selection. In MBS, six of the eight representative studies in **Table 4** were retrospective, and neither prospective report demonstrated improved patient outcomes resulting from AI-guided implementation. One prospective study evaluated predefined clinician-designed alerts without a machine-learning prediction model, whereas the other developed and internally validated a model without prospectively testing model-guided care. Thus, none of the eight studies established clinical effectiveness. For rare adverse events, moderate AUROC may still yield poor positive predictive value or net benefit, further emphasising the need for prospective impact studies rather than additional retrospective performance analyses. LLMs are a separate category: they may support education and drafting, yet low real-world procedure concordance, variable readability, hallucinations and unstable outputs preclude autonomous use in treatment or surgical decisions. Five cross-cutting gaps recur: emphasis on performance rather than long-term outcomes; inadequate external validation and calibration; unclear action thresholds and workflow effects; limited generalisability across populations and health systems; and insufficient assessment of safety, privacy, fairness, alert burden, accountability and post-deployment monitoring. AI should therefore be positioned as an adjunct within clinician-led, multidisciplinary care. Clinical translation requires evidence of net benefit, not merely technical novelty or high apparent accuracy. Domain-level evidence profiles, currently supported roles and priority evidence gaps are summarised in **Table 5**.

**Table 5:** Domain-level evidence profiles, currently supported clinical roles and priority evidence gaps for AI applications in obesity care.

Domain	Clinical or clinically relevant evidence	External validation	Prospective workflow or patient-outcome evidence	Currently supported clinical role and priority evidence gap
Explicitly AI-enabled lifestyle interventions and contextual digital-care evidence (5, 21–24)	Direct AI-specific patient-level evidence is limited to the AI-DPP pragmatic noninferiority trial and the short-term, single-arm eTRIP study. Oviva Direkt and zanadio did not explicitly evaluate an AI component, while Lee et al. primarily studied type 2 diabetes and used HbA1c as the primary endpoint. These three studies therefore provide contextual digital-care or adjacent metabolic-care evidence rather than evidence of an AI-specific therapeutic effect in obesity.	Cross-platform, cross-population and cross-health-system replication remains limited, and no component-level evaluation has isolated the independent therapeutic contribution of AI.	AI-DPP provides 12-month randomised evidence for the complete AI-led delivery model but does not demonstrate clinical superiority over human coaching or an isolated AI effect. eTRIP provides only 1-week feasibility and behavioural data. Prospective outcomes from Oviva Direkt and Lee et al. support digital-care or diabetes-management delivery rather than AI-specific effectiveness in obesity, while zanadio primarily demonstrates programme reach and infrastructure.	Currently supported role: supervised delivery within multicomponent lifestyle-care pathways. An independent AI-specific therapeutic benefit has not been established. Priorities include longer pragmatic trials, component-level evaluation, safety, cost-effectiveness and digital equity.
Chatbots and generative AI (8, 10, 25–30, 56–60)	Predominantly simulations, proof-of-concept studies and small short-term evaluations.	Minimal; performance is model-, version- and prompt-dependent.	No convincing evidence of durable patient benefit or safe unsupervised treatment decisions.	Currently supported role: clinician-reviewed education, reminders and drafting rather than unsupervised treatment or surgical decision-making. Priorities include reproducibility, citation accuracy, readability, human oversight and prospective safety evaluation.
Pharmacotherapy-related AI (6, 34–53)	Includes preclinical discovery, hypothesis-generating text analyses and candidate responder stratification.	No independently validated AI prescribing system identified.	No trial showing improved outcomes from AI-guided drug selection or dose adjustment.	Currently supported role: preclinical discovery, signal detection and hypothesis generation rather than routine AI-guided prescribing. Priorities include cross-ancestry validation, calibration, interpretability and comparative clinical trials.
Bariatric prediction, computer vision and weight-trajectory modelling (7, 68–79)	Large retrospective registries and technical studies predominate; some trajectory models have international external testing.	Variable; many models use internal or temporal validation only.	Rarely evaluated as a live workflow intervention; improved patient outcomes have not been established.	Currently supported role: supervised research and risk-identification support where locally validated, rather than autonomous clinical decision-making. Priorities include geographical validation, calibration, actionable thresholds, decision curves and implementation trials.
Postoperative digital monitoring (80, 81)	Two prospective single-centre reports each involved 104 patients: one evaluated predefined clinician-designed alerts without a machine-learning prediction model, and the other developed and internally validated a dynamic machine-learning complication model.	Independent external validation is absent.	Alert–outcome associations and internal model performance were reported, but neither study prospectively tested whether model-guided care reduced complications, emergency-department visits, readmissions or clinical workload.	Currently supported role: prospective feasibility monitoring with predefined clinician review and escalation pathways. AI-specific clinical effectiveness has not been demonstrated. Priorities include external validation, alert-burden analysis, explicit escalation pathways and randomised workflow evaluation.

AI, artificial intelligence.

The contribution of this review lies in its analytical lens and output rather than in identifying previously unreviewed AI modalities. Earlier obesity-specific reviews mapped AI-supported behavioural self-regulation (83), chatbot or conversational-agent design and short-term effectiveness (84), broad AI applications in obesity prediction and management (85), and perioperative applications in bariatric surgery (12). A recent systematic review separately synthesised 33 studies of LLMs in medical and surgical obesity care (10). FUTURE-AI addresses a complementary, disease-agnostic question by defining 30 best practices across fairness, universality, traceability, usability, robustness and explainability, as well as general requirements spanning design, regulation, deployment and monitoring (86). The present review applies a consistent obesity-specific readiness lens across these otherwise separate evidence streams. In particular, it links each application to an intended clinical action and the consequences of error, distinguishes multicomponent digital-intervention effects from an independently demonstrated AI effect, and identifies whether external validation, calibration, clinical utility, workflow integration, human oversight and prospective patient benefit have been demonstrated. The resulting descriptive evidence-profile matrix therefore complements rather than replaces general frameworks such as FUTURE-AI.

### 6.1 Limitations

This was a SANRA-informed structured narrative review rather than a systematic review or meta-analysis. Although the search, eligibility criteria and screening process were described, we did not perform formal PRISMA flow reporting, GRADE assessment, quantitative pooling or standardized study-level risk-of-bias scoring. The synthesis is therefore representative and clinically oriented rather than exhaustive (15). Database coverage, indexing and Google Scholar ranking may have omitted recent or non-indexed studies. The field is also rapidly changing, particularly for commercial digital platforms and LLM versions, limiting reproducibility and temporal stability. Substantial heterogeneity prevented direct comparison: digital studies evaluated multicomponent interventions; drug-discovery studies were largely preclinical; pharmacovigilance studies used unverified online text; surgical models were often retrospective or registry-based; and LLM studies used simulated questions or retrospective case comparisons. Domain-specific limitations include rare-event class imbalance, missing data, coding error, limited calibration and external validation in surgical models; inability to isolate AI effects in digital interventions; and uncertain representativeness and causal interpretation in social-media analyses. LLM studies additionally vary in model version, prompts, repeated runs and evaluation methods and remain vulnerable to hallucination and incorrect citation. Evidence regarding fairness, privacy, safety, cost-effectiveness, clinician workload and long-term monitoring remains limited.

### 6.2 Future research agenda

Future research should move from demonstrating prediction or content generation to showing improvement in long-term weight, metabolic outcomes, complications, readmissions, quality of life, workload, cost-effectiveness and health equity in real clinical pathways. The design, reporting and appraisal of future studies should use domain-appropriate frameworks: CONSORT-AI for reports of completed AI intervention trials, SPIRIT-AI for AI clinical-trial protocols, DECIDE-AI for early live clinical evaluations of AI decision-support systems, TRIPOD+AI and PROBAST+AI for prediction-model studies, and STARD-AI for AI-based diagnostic-accuracy studies. These frameworks should guide description of intended use, inputs and outputs, human-AI interaction, validation, calibration, error analysis, workflow integration and safety. Digital lifestyle trials should be longer, pragmatic and demographically representative, clearly distinguishing consumer applications, prescription digital therapeutics, automated coaching and hybrid care. They should report the active intervention components, model updates, human oversight, retention, discontinuation, safety, long-term weight maintenance, cardiometabolic outcomes, burden, cost and digital equity. Precision pharmacotherapy research should develop externally validated, calibrated and interpretable models using clinically available phenotype, early response, tolerability, behavioural and preference data. Prospective trials must test whether AI-guided medication selection or dose adjustment improves efficacy, adherence, safety and cost compared with clinician-led care. Drug-discovery studies should transparently report training data, molecular libraries, model architecture, experimental validation, pharmacokinetics and toxicity and should not claim clinical effectiveness before human trials. Pharmacovigilance studies should link online signals to clinical, prescription, claims and formal adverse-event data and report sampling, deduplication, bot filtering, annotation, validation and human adjudication. For bariatric prediction models, external validation should be geographically and temporally independent. Studies should report discrimination, calibration intercept and slope, Brier score when appropriate, decision curves, positive and negative predictive values, clinically actionable thresholds and subgroup performance, and should test whether linked actions reduce complications or resource use. Computer-vision research should separate patients, surgeons and centres between training and testing and report phase-specific performance, video quality, annotation standards, confusion matrices and error cases. The clinically relevant endpoints are improved training, standardised feedback, operative quality and safety, not step-recognition accuracy alone. Postoperative digital follow-up should be studied as a workflow intervention. Trials should report alert thresholds, frequency, false-positive and false-negative burden, response times, escalation pathways, adherence, attrition, clinician workload and whether monitoring reduces delay, emergency visits, readmissions or unnecessary appointments. LLM studies should report model version, access date, prompts, retrieval sources, repeated runs, output variability, evaluator expertise, inter-rater agreement, hallucination and citation accuracy, readability, patient understanding and review burden. High-risk treatment, procedure and triage tasks should remain clinician supervised until prospectively validated. Across all domains, implementation research should assess automation bias, recommendation acceptance or override, accountability, unintended consequences and post-deployment drift. Equity, privacy, cybersecurity, governance and sustainable payment models should be incorporated from study design onward.

### 6.3 Regulatory and governance requirements for clinical deployment

Regulatory obligations should be determined by intended purpose, clinical claims and risk, rather than by whether a product is marketed as “AI.” Systems that select anti-obesity medication, triage postoperative symptoms, predict complications or influence bariatric procedure choice may constitute software as a medical device or an AI-enabled medical-device function and therefore require the applicable premarket review or conformity assessment. In the United States, qualifying functions are regulated through existing FDA device pathways and planned post-authorisation modifications may be addressed through an FDA-authorised predetermined change control plan (PCCP) included in the marketing submission. In the European Union, AI that is itself a medical device, or a safety component of one, is considered high risk under the AI Act when the associated product requires third-party conformity assessment; relevant Medical Device Regulation or *In Vitro* Diagnostic Medical Device Regulation obligations also remain applicable. International guidance further emphasises clear intended use, representative data, independent testing, cybersecurity, human–AI interaction and monitoring across the total product lifecycle (86, 87).

Regulatory authorisation alone is insufficient for safe deployment. Obesity-care systems can combine longitudinal weight, dietary, behavioural, mental-health, wearable, medication, genetic and perioperative data. Institutional governance should therefore define lawful data provenance and use, minimum-necessary access, data retention, security, vendor and model-version responsibility, local validation and subgroup performance by sex, age, ethnicity, socioeconomic position, disability, digital literacy and genetic ancestry where relevant. Patients should receive proportionate information about the AI system’s role, data use, limitations and accountable clinician; when AI materially influences high-risk decisions, consent procedures should follow local law and professional guidance. Medication selection, surgical candidacy, procedure choice and urgent postoperative triage should remain clinician supervised, with explicit override and escalation pathways. After deployment, organisations should retain audit logs and monitor data and performance drift, calibration, false alerts, adverse events, subgroup harm, cybersecurity incidents, workload and patient outcomes. Predefined thresholds should trigger investigation and, where appropriate, suspension, controlled retraining and revalidation, regulatory reassessment, or withdrawal.

### 6.4 Healthcare resource utilisation, reimbursement and economic scalability

AI-supported obesity care may alter healthcare resource utilisation by automating enrolment, risk stratification, personalised feedback and routine monitoring, while directing clinician attention towards patients who require treatment escalation. In a pragmatic randomised trial, initiation was higher following referral to an AI-led Diabetes Prevention Program than to a human coach-led programme (93.4% vs 82.7%), and the AI-led intervention was noninferior for the prespecified composite clinical outcome (5). These findings support the potential for AI to extend programme capacity where trained coaching personnel are limited; however, the trial did not include a formal cost-effectiveness, budget-impact or healthcare-utilisation analysis.

Evidence from adjacent digital weight-management programmes remains mixed. In a six-month propensity-matched US claims study, participation in a commercial smartphone-based programme was associated with 3.2 fewer outpatient visits, 0.34 fewer emergency-department visits, 0.25 fewer inpatient visits and USD 831 lower healthcare costs per participant (88). Conversely, another propensity-matched health-system study found increased specialty-care, urgent-care, electronic and obesity-related outpatient contacts during the second year after participation in a lifestyle programme (89). Such increases may reflect appropriate detection, treatment initiation and preventive follow-up rather than inefficiency. Importantly, these studies were nonrandomised and remained susceptible to self-selection and residual confounding despite propensity-score matching. They evaluated multicomponent interventions and, in the former study, involved investigators employed or contracted by the programme provider. They therefore do not establish that the AI component itself reduces resource use.

Long-term economic estimates should be interpreted with similar caution. A remotely delivered, DPP-like behavioural programme for older adults projected cumulative medical savings of USD 1,720–1,770 per participant at three years and USD 11,550–14,200 at ten years (90). However, these estimates were generated using a Markov microsimulation based on short-term weight loss rather than observed long-term expenditure and were sensitive to assumptions regarding weight regain and programme costs. Although automation may reduce the marginal delivery cost of adding participants, implementation also entails substantial fixed and recurrent costs, including software and device acquisition, electronic health record integration, cybersecurity and data governance, staff training, human review and escalation, model monitoring, recalibration, updates and vendor management. Alert burden, low engagement or the need to support patients with limited digital access may further increase staffing requirements. Consequently, scalability should be evaluated using the cost per additional successfully treated patient—not merely the number of users enrolled—and may be unfavourable at low volumes or when human escalation rates are high.

Reimbursement is jurisdiction- and product-specific and cannot be inferred from technical performance alone. A US payer Delphi study found that digital products were more likely to be considered for coverage when they had undergone regulatory review, required a prescription, treated rather than only monitored a condition, had low clinical opportunity cost and could improve population-health metrics (91). A comparison of health technology assessment frameworks similarly showed that requirements vary across countries; at the time of that review, budget-impact analysis was expected across the identified digital-health evidence tiers, while cost-utility analysis was recommended for technologies with high financial commitment (92). In obesity care, the economic effect of an AI-supported pathway will also depend on the interventions it recommends. High and continuing expenditure on GLP-1–based medications creates major affordability and coverage challenges (93), and a US cohort study found that two-year adjusted costs were higher for GLP-1 receptor agonist treatment than for metabolic and bariatric surgery in class II or III obesity, largely because of sustained pharmacy expenditure (94). AI cannot itself remove formulary restrictions, medication prices, procedural capacity constraints or out-of-pocket costs and could worsen inequity if it preferentially recommends options that are inaccessible to disadvantaged patients.

Future pragmatic studies should therefore evaluate the incremental value of AI against a clearly defined human-led or non-AI digital comparator. Recommended outcomes include programme and implementation costs, clinician and administrative time, inpatient, outpatient and medication utilisation, patient out-of-pocket expenditure, cost per quality-adjusted life-year, budget impact, return on investment, treatment discontinuation and equity-stratified results. Sensitivity analyses should address programme scale, participant retention, human-escalation rates, model-update costs and changes in medication prices.

## 7 Conclusions

AI research is producing potentially useful tools for obesity care, but evidence supporting routine clinical adoption remains limited and differs substantially across application domains. Direct patient-level evidence for explicitly AI-enabled obesity interventions remains limited. Broader digital, automated and hybrid-care programmes have stronger patient-level evidence and may improve access, engagement and short-term weight or metabolic outcomes, but their benefits should be attributed to the complete behavioural and digital-care model rather than to AI alone. In pharmacotherapy, near-term applications are most credible in preclinical discovery and supplementary patient-experience or safety-signal analysis. The evidence identified in this review did not validate AI-guided medication selection for routine care. In MBS, prediction, computer vision, remote monitoring and trajectory modelling show technical promise but generally lack sufficient external validation and prospective evidence of clinical net benefit. LLMs may assist education, information organisation and documentation, but hallucination, variable reproducibility and low concordance with complex clinical decisions require strict human oversight. Across domains, AI should support rather than replace clinicians and multidisciplinary teams. Reliable translation will depend on transparent reporting, independent validation, calibration, actionable thresholds, prospective workflow evaluation, continuous safety and fairness monitoring, data governance and evidence of improved patient-centred outcomes and cost-effectiveness.
