# Supplementary material for: Artificial intelligence in obesity management: clinical evidence, translational gaps, and implementation priorities—a structured narrative review
Source: Front Endocrinol (Lausanne). 2026 Sep 3;17:1955223. doi: 10.3389/fendo.2026.1955223 (PMC13581758; doi:10.3389/fendo.2026.1955223)
Supplement: Supplementary file 1 [file SupplementaryFile1.docx]

# Supplementary Search Strategies

## S1. PubMed/MEDLINE

**Search date:** 1 June 2026
**Coverage:** 1 January 2015–1 June 2026
**Filters:** English language; no article-type, human-only, age, sex, or full-text filter.

### S1.1 Principal search

(

"Obesity"[Mesh]

OR "Overweight"[Mesh]

OR "Weight Loss"[Mesh]

OR "Bariatric Surgery"[Mesh]

OR obes*[tiab]

OR overweight[tiab]

OR adiposity[tiab]

OR "weight loss"[tiab]

OR "weight reduction"[tiab]

OR "weight management"[tiab]

OR "body mass index"[tiab]

OR BMI[tiab]

OR antiobes*[tiab]

OR "anti-obesity medication"[tiab]

OR "anti-obesity medications"[tiab]

OR "anti-obesity agent"[tiab]

OR "anti-obesity agents"[tiab]

OR "bariatric surgery"[tiab]

OR "bariatric procedure"[tiab]

OR "bariatric procedures"[tiab]

OR "metabolic surgery"[tiab]

OR "sleeve gastrectomy"[tiab]

OR "gastric bypass"[tiab]

OR "Roux-en-Y gastric bypass"[tiab]

)

AND

(

"Artificial Intelligence"[Mesh]

OR "Machine Learning"[Mesh]

OR "Deep Learning"[Mesh]

OR "Natural Language Processing"[Mesh]

OR "Neural Networks, Computer"[Mesh]

OR "Decision Support Systems, Clinical"[Mesh]

OR "Computer Vision"[Mesh]

OR "artificial intelligence"[tiab]

OR "machine learning"[tiab]

OR "deep learning"[tiab]

OR "natural language processing"[tiab]

OR "computer vision"[tiab]

OR "neural network"[tiab]

OR "neural networks"[tiab]

OR "artificial neural network"[tiab]

OR "artificial neural networks"[tiab]

OR "convolutional neural network"[tiab]

OR "convolutional neural networks"[tiab]

OR "random forest"[tiab]

OR "random forests"[tiab]

OR "support vector machine"[tiab]

OR "support vector machines"[tiab]

OR "gradient boosting"[tiab]

OR XGBoost[tiab]

OR "predictive model"[tiab]

OR "predictive models"[tiab]

OR "prediction model"[tiab]

OR "prediction models"[tiab]

OR "predictive algorithm"[tiab]

OR "predictive algorithms"[tiab]

OR "risk prediction"[tiab]

OR "clinical decision support"[tiab]

OR "recommendation system"[tiab]

OR "recommendation systems"[tiab]

OR "recommender system"[tiab]

OR "recommender systems"[tiab]

OR "image recognition"[tiab]

OR "food image recognition"[tiab]

OR "automated image analysis"[tiab]

OR "video analysis"[tiab]

OR "phase recognition"[tiab]

OR "workflow recognition"[tiab]

OR "natural language model"[tiab]

OR "large language model"[tiab]

OR "large language models"[tiab]

OR LLM[tiab]

OR LLMs[tiab]

OR ChatGPT[tiab]

OR "GPT-3.5"[tiab]

OR "GPT-4"[tiab]

OR Bard[tiab]

OR Gemini[tiab]

OR BERT[tiab]

OR transformer*[tiab]

OR "generative AI"[tiab]

OR "generative artificial intelligence"[tiab]

OR "foundation model"[tiab]

OR "foundation models"[tiab]

OR "retrieval-augmented generation"[tiab]

OR "retrieval augmented generation"[tiab]

OR RAG[tiab]

OR chatbot*[tiab]

OR "conversational agent"[tiab]

OR "conversational agents"[tiab]

OR "virtual coach"[tiab]

OR "virtual coaches"[tiab]

OR

(

(

"digital health"[tiab]

OR "digital intervention"[tiab]

OR "digital interventions"[tiab]

OR "digital therapeutic"[tiab]

OR "digital therapeutics"[tiab]

OR "mobile health"[tiab]

OR mHealth[tiab]

OR "mobile application"[tiab]

OR "mobile applications"[tiab]

OR "mobile app"[tiab]

OR "mobile apps"[tiab]

OR wearable*[tiab]

OR telehealth[tiab]

OR telemedicine[tiab]

OR "remote monitoring"[tiab]

)

AND

(

algorithm*[tiab]

OR adaptiv*[tiab]

OR personaliz*[tiab]

OR personalis*[tiab]

OR automat*[tiab]

OR intelligent[tiab]

)

)

)

AND English[lang]

AND ("2015/01/01"[Date - Publication] : "2026/07/22"[Date - Publication])

### S1.2 Medication-focused supplementary search

(

"Obesity"[Mesh]

OR obes*[tiab]

OR overweight[tiab]

OR "weight loss"[tiab]

OR "weight management"[tiab]

OR antiobes*[tiab]

)

AND

(

semaglutide[tiab]

OR tirzepatide[tiab]

OR liraglutide[tiab]

OR "GLP-1"[tiab]

OR "GLP-1 receptor agonist"[tiab]

OR "GLP-1 receptor agonists"[tiab]

OR "glucagon-like peptide-1"[tiab]

OR "GIP/GLP-1"[tiab]

OR incretin*[tiab]

OR pharmacotherap*[tiab]

OR pharmacovigilance[tiab]

OR "drug discovery"[tiab]

OR "drug development"[tiab]

OR "drug design"[tiab]

OR "responder stratification"[tiab]

OR "treatment response prediction"[tiab]

)

AND

(

"Artificial Intelligence"[Mesh]

OR "Machine Learning"[Mesh]

OR "Natural Language Processing"[Mesh]

OR "artificial intelligence"[tiab]

OR "machine learning"[tiab]

OR "deep learning"[tiab]

OR "natural language processing"[tiab]

OR "neural network"[tiab]

OR "neural networks"[tiab]

OR BERT[tiab]

OR transformer*[tiab]

OR "large language model"[tiab]

OR "large language models"[tiab]

OR "generative AI"[tiab]

OR "sentiment analysis"[tiab]

OR "topic modelling"[tiab]

OR "topic modeling"[tiab]

)

AND English[lang]

AND ("2015/01/01"[Date - Publication] : "2026/07/22"[Date - Publication])

## S2. Embase

**Platform:** Embase.com
**Search date:** 1 June 2026
**Coverage:** 2015–1 June 2026
**Filters:** English language; no human-only, source-type, or article-type restriction.

### S2.1 Principal search

(

'obesity'/exp

OR 'overweight'/exp

OR 'weight reduction'/exp

OR 'bariatric surgery'/exp

OR obes*:ti,ab,kw

OR overweight:ti,ab,kw

OR adiposity:ti,ab,kw

OR 'weight loss':ti,ab,kw

OR 'weight reduction':ti,ab,kw

OR 'weight management':ti,ab,kw

OR 'body mass index':ti,ab,kw

OR BMI:ti,ab,kw

OR antiobes*:ti,ab,kw

OR 'anti-obesity medication':ti,ab,kw

OR 'anti-obesity medications':ti,ab,kw

OR 'bariatric surgery':ti,ab,kw

OR 'bariatric procedure':ti,ab,kw

OR 'bariatric procedures':ti,ab,kw

OR 'metabolic surgery':ti,ab,kw

OR 'sleeve gastrectomy':ti,ab,kw

OR 'gastric bypass':ti,ab,kw

OR 'Roux-en-Y gastric bypass':ti,ab,kw

)

AND

(

'artificial intelligence'/exp

OR 'machine learning'/exp

OR 'deep learning'/exp

OR 'natural language processing'/exp

OR 'neural network'/exp

OR 'computer vision'/exp

OR 'clinical decision support system'/exp

OR 'artificial intelligence':ti,ab,kw

OR 'machine learning':ti,ab,kw

OR 'deep learning':ti,ab,kw

OR 'natural language processing':ti,ab,kw

OR 'computer vision':ti,ab,kw

OR 'neural network':ti,ab,kw

OR 'neural networks':ti,ab,kw

OR 'artificial neural network':ti,ab,kw

OR 'artificial neural networks':ti,ab,kw

OR 'convolutional neural network':ti,ab,kw

OR 'convolutional neural networks':ti,ab,kw

OR 'random forest':ti,ab,kw

OR 'random forests':ti,ab,kw

OR 'support vector machine':ti,ab,kw

OR 'support vector machines':ti,ab,kw

OR 'gradient boosting':ti,ab,kw

OR xgboost:ti,ab,kw

OR 'predictive model':ti,ab,kw

OR 'predictive models':ti,ab,kw

OR 'prediction model':ti,ab,kw

OR 'prediction models':ti,ab,kw

OR 'risk prediction':ti,ab,kw

OR 'clinical decision support':ti,ab,kw

OR 'recommendation system':ti,ab,kw

OR 'recommendation systems':ti,ab,kw

OR 'recommender system':ti,ab,kw

OR 'recommender systems':ti,ab,kw

OR 'image recognition':ti,ab,kw

OR 'food image recognition':ti,ab,kw

OR 'video analysis':ti,ab,kw

OR 'phase recognition':ti,ab,kw

OR 'workflow recognition':ti,ab,kw

OR 'large language model':ti,ab,kw

OR 'large language models':ti,ab,kw

OR LLM:ti,ab,kw

OR LLMs:ti,ab,kw

OR chatgpt:ti,ab,kw

OR 'GPT-3.5':ti,ab,kw

OR 'GPT-4':ti,ab,kw

OR bard:ti,ab,kw

OR gemini:ti,ab,kw

OR bert:ti,ab,kw

OR transformer*:ti,ab,kw

OR 'generative AI':ti,ab,kw

OR 'generative artificial intelligence':ti,ab,kw

OR 'foundation model':ti,ab,kw

OR 'foundation models':ti,ab,kw

OR 'retrieval-augmented generation':ti,ab,kw

OR 'retrieval augmented generation':ti,ab,kw

OR chatbot*:ti,ab,kw

OR 'conversational agent':ti,ab,kw

OR 'conversational agents':ti,ab,kw

OR 'virtual coach':ti,ab,kw

OR 'virtual coaches':ti,ab,kw

OR

(

(

'digital health':ti,ab,kw

OR 'digital intervention':ti,ab,kw

OR 'digital interventions':ti,ab,kw

OR 'digital therapeutic':ti,ab,kw

OR 'digital therapeutics':ti,ab,kw

OR 'mobile health':ti,ab,kw

OR mhealth:ti,ab,kw

OR 'mobile application':ti,ab,kw

OR 'mobile applications':ti,ab,kw

OR wearable*:ti,ab,kw

OR telehealth:ti,ab,kw

OR telemedicine:ti,ab,kw

OR 'remote monitoring':ti,ab,kw

)

AND

(

algorithm*:ti,ab,kw

OR adaptiv*:ti,ab,kw

OR personaliz*:ti,ab,kw

OR personalis*:ti,ab,kw

OR automat*:ti,ab,kw

OR intelligent:ti,ab,kw

)

)

)

**Applied limits**

Publication year: 2015–2026

Final search date: 1 June 2026

Language: English

Source type: No restriction

Human studies: No restriction

Article type: No restriction

## S3. Scopus

**Search date:** 1 June 2026
**Coverage:** 2015–2026
**Filters:** English language; no document-type, subject-area, or open-access restriction.

### S3.1 Principal search

TITLE-ABS-KEY(

(

obes*

OR overweight

OR adiposity

OR "weight loss"

OR "weight reduction"

OR "weight management"

OR "body mass index"

OR BMI

OR antiobes*

OR "anti-obesity medication"

OR "anti-obesity medications"

OR "bariatric surgery"

OR "bariatric procedure"

OR "bariatric procedures"

OR "metabolic surgery"

OR "sleeve gastrectomy"

OR "gastric bypass"

OR "Roux-en-Y gastric bypass"

)

AND

(

"artificial intelligence"

OR "machine learning"

OR "deep learning"

OR "natural language processing"

OR "computer vision"

OR "neural network"

OR "neural networks"

OR "artificial neural network"

OR "artificial neural networks"

OR "convolutional neural network"

OR "convolutional neural networks"

OR "random forest"

OR "random forests"

OR "support vector machine"

OR "support vector machines"

OR "gradient boosting"

OR XGBoost

OR "predictive model"

OR "predictive models"

OR "prediction model"

OR "prediction models"

OR "predictive algorithm"

OR "predictive algorithms"

OR "risk prediction"

OR "clinical decision support"

OR "recommendation system"

OR "recommendation systems"

OR "recommender system"

OR "recommender systems"

OR "image recognition"

OR "food image recognition"

OR "automated image analysis"

OR "video analysis"

OR "phase recognition"

OR "workflow recognition"

OR "large language model"

OR "large language models"

OR LLM

OR LLMs

OR ChatGPT

OR "GPT-3.5"

OR "GPT-4"

OR Bard

OR Gemini

OR BERT

OR transformer*

OR "generative AI"

OR "generative artificial intelligence"

OR "foundation model"

OR "foundation models"

OR "retrieval-augmented generation"

OR "retrieval augmented generation"

OR chatbot*

OR "conversational agent"

OR "conversational agents"

OR "virtual coach"

OR "virtual coaches"

OR

(

(

"digital health"

OR "digital intervention"

OR "digital interventions"

OR "digital therapeutic"

OR "digital therapeutics"

OR "mobile health"

OR mHealth

OR "mobile application"

OR "mobile applications"

OR wearable*

OR telehealth

OR telemedicine

OR "remote monitoring"

)

AND

(

algorithm*

OR adaptiv*

OR personaliz*

OR personalis*

OR automat*

OR intelligent

)

)

)

)

AND PUBYEAR > 2014

AND PUBYEAR < 2027

AND LIMIT-TO(LANGUAGE, "English")

### S3.2 Medication-focused supplementary search

TITLE-ABS-KEY(

(

obes*

OR overweight

OR "weight loss"

OR "weight management"

OR antiobes*

)

AND

(

semaglutide

OR tirzepatide

OR liraglutide

OR "GLP-1"

OR "GLP-1 receptor agonist"

OR "GLP-1 receptor agonists"

OR "glucagon-like peptide-1"

OR "GIP/GLP-1"

OR incretin*

OR pharmacotherap*

OR pharmacovigilance

OR "drug discovery"

OR "drug development"

OR "drug design"

OR "responder stratification"

OR "treatment response prediction"

)

AND

(

"artificial intelligence"

OR "machine learning"

OR "deep learning"

OR "natural language processing"

OR "neural network"

OR BERT

OR transformer*

OR "large language model"

OR "large language models"

OR "generative AI"

OR "sentiment analysis"

OR "topic modelling"

OR "topic modeling"

)

)

AND PUBYEAR > 2014

AND PUBYEAR < 2027

AND LIMIT-TO(LANGUAGE, "English")

## S4. ScienceDirect

**Search date:** 1 June 2026
**Coverage:** 2015–2026
**Search field:** Title, abstract, or author-specified keywords
**Filters:** Journals; English-language screening; no open-access restriction.

### S4.1 General AI and obesity

(obesity OR overweight OR "weight management")

AND

("artificial intelligence" OR "machine learning" OR "deep learning")

### S4.2 Predictive models in obesity

(obesity OR overweight OR "weight loss")

AND

("predictive model" OR "neural network" OR "risk prediction")

### S4.3 AI-enabled digital lifestyle interventions

(obesity OR "weight management")

AND

("digital therapeutic" OR mHealth OR wearable)

AND

(adaptive OR personalized OR automated)

### S4.4 Conversational agents and weight management

("weight loss" OR "weight management" OR obesity)

AND

(chatbot OR "conversational agent" OR "virtual coach")

### S4.5 Computer vision and dietary assessment

(obesity OR overweight OR "weight management")

AND

("computer vision" OR "food image recognition" OR "image recognition")

### S4.6 Generative AI and obesity

(obesity OR "weight management")

AND

("large language model" OR ChatGPT OR "generative AI")

### S4.7 AI and anti-obesity pharmacotherapy

(obesity OR "anti-obesity medication")

AND

("machine learning" OR "natural language processing")

AND

(semaglutide OR tirzepatide OR "GLP-1")

### S4.8 AI-assisted obesity drug discovery

(obesity OR antiobesity)

AND

("artificial intelligence" OR "machine learning")

AND

("drug discovery" OR "drug design" OR QSAR)

### S4.9 General AI in bariatric surgery

("bariatric surgery" OR "metabolic surgery")

AND

("artificial intelligence" OR "machine learning" OR "deep learning")

### S4.10 Computer vision in bariatric surgery

("bariatric surgery" OR "metabolic surgery")

AND

("computer vision" OR "phase recognition" OR "video analysis")

### S4.11 Bariatric risk and outcome prediction

("bariatric surgery" OR "metabolic surgery")

AND

("risk prediction" OR readmission OR complication)

AND

("machine learning" OR "artificial intelligence")

### S4.12 LLMs and bariatric surgery

("bariatric surgery" OR "metabolic surgery")

AND

(ChatGPT OR "large language model" OR "generative AI")

**Applied limits**

Publication years: 2015–2026

Content type: Journals

Access type: No restriction

Article type: No search-stage restriction

Final search date: 1 June 2026

## S5. Google Scholar

**Search date:** 1 June 2026
**Coverage:** Custom year range 2015–2026
**Sort order:** Relevance
**Limits:** First 200 results per query; patents and citation-only records excluded.

### S5.1 Lifestyle, digital interventions, and weight management

(obesity OR overweight)

("artificial intelligence" OR "machine learning" OR chatbot OR "digital therapeutic")

("weight loss" OR "weight management")

### S5.2 Pharmacotherapy, drug development, and pharmacovigilance

obesity

("machine learning" OR "artificial intelligence" OR "natural language processing")

(semaglutide OR tirzepatide OR "GLP-1" OR pharmacovigilance OR "drug discovery")

### S5.3 Metabolic and bariatric surgery

("bariatric surgery" OR "metabolic surgery")

("artificial intelligence" OR "machine learning" OR "deep learning" OR "computer vision")

### S5.4 LLMs, generative AI, and conversational agents

(obesity OR "bariatric surgery")

(ChatGPT OR "large language model" OR chatbot OR "conversational agent" OR "generative AI")

**Applied Google Scholar limits**

Custom year range: 2015–2026

Sort order: Relevance

Results screened: First 200 results per query

Patents: Excluded

Citation-only records: Excluded

Maximum displayed screening set:

4 queries × 200 results = 800 records before deduplication
